# Phytoestrogen Signal Pathways and Estrogen Signaling in Ovarian Cancer: A Narrative Review

**DOI:** 10.1002/ptr.70013

**Published:** 2025-07-09

**Authors:** Ozgur Kutuk, Ayse Kaplan

**Affiliations:** ^1^ Molecular Biology and Genetics, Bioengineering Program, Faculty of Engineering and Natural Sciences Sabanci University Istanbul Tuzla Turkey; ^2^ Department of Molecular Biology, Faculty of Science Eskisehir Technical University Eskisehir Tepebasi Turkey

**Keywords:** estrogens receptor, GPER, ovarian cancer, phytoestrogens, signal pathway

## Abstract

Ovarian cancer (OC) is the second most common gynecological cancer and the leading cause of death from gynecological malignancies. Ovarian cancer mortality rate ranks fifth among cancer‐related deaths in Western societies. Hence, novel preventive and therapeutic ways are still in great demand to reduce the incidence and mortality rate of ovarian cancer. Phytoestrogens, referred to as dietary estrogens, provide benefits to all mammals, including humans. Research indicates that phytoestrogens may be possible hormonal treatment options for ovarian cancer patients. They are non‐steroidal plant compounds that undergo metabolism to produce compounds structurally and functionally related to ovarian and placental estrogens. Some studies suggest that estrogen receptors (ER‐α and ER‐β) and G protein‐coupled estrogen receptor (GPER) are potential targets for ovarian cancer prevention and treatment. Current studies indicate multiple signal pathways of phytoestrogens in the management of ovarian cancer. Even so, literature suggests that the signaling mechanisms in ovarian cancer and the signaling mechanisms of phytoestrogens are still not exactly understood. With this, phytoestrogens may act on multiple signaling pathways such as ER (endoplasmic reticulum)‐dependent signaling, GnRH receptor, FSH or LH receptors and hormones, and GFR, which help to regulate the expression of AKT, RAS, RAF, Caspase‐3, NF‐kB, and Bcl‐2. In summary, this narrative review discusses the possible targets of phytoestrogens in ovarian cancer and sheds a light on improving novel phytoestrogens‐based dietary supplements against ovarian cancers.

## Graphical abstract

Phytoestrogen signal pathways targeted in ovarian cancer.

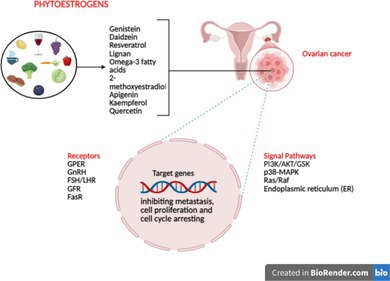

**Abbreviations**
AKT: protein kinase B
ATF3: activating transcription factor 3
ATF6: activating transcription factor 6
Bad: Bcl‐2 associated agonist of cell death
Bax: Bcl‐2 associated x‐protein
Bcl‐2: B‐cell lymphoma 2
Bid: BH3‐interacting domain death agonist
Caspase‐3: cysteine–aspartic acid protease‐3
Caspase‐4: cysteine–aspartic acid protease‐4
Caspase‐8: cysteine–aspartic acid protease‐8
Caspase‐9: cysteine–aspartic acid protease‐9
Cdc2: cell division control 2
Cdc25C: cell division cycle 25C
CDK4: cyclin‐dependent kinase 4
Chk2: checkpoint‐kinase‐2
COX: cyclooxygenase
Cyto‐c: cytochrome c
DR5: death receptor 5
E2: estradiol, 17β‐estradiol
EGFR: epidermal growth factor receptor
ER: endoplasmic reticulum
ERK: extracellular signal‐regulated kinase
ERK‐1: extracellular signal‐regulated kinase1
ERs: estrogen receptors
ER‐α: estrogen receptor α
ER‐β: estrogen receptor β
ESRRA: estrogen related receptor alpha
FAD: flavin adenine dinucleotide
FADD: Fas‐associated death domain protein
Fak: focal adhesion kinase
FOXL‐2: forkhead box protein L2
FSH: follicle‐stimulating hormone
GFR: glomerular filtration rate
GnRH: gonadotropin‐releasing hormone
GPER: G protein‐coupled estrogen receptor
GREB1: growth regulation by estrogen in breast cancer
GRP78: glucose regulated protein 78
GSK: glycogen synthase kinase
HDM2: human double minute 2
HIF‐1: hypoxia‐inducible factor
Id1: inhibitor of differentiation or DNA binding protein 1
IL‐6: interleukin‐6
IRE‐1: inositol‐requiring enzyme‐1
LC3II: light chain 3‐II
LH: luteinizing hormone
MAPK: mitogen‐activated protein kinase
MMP: matrix metalloproteinase
mTOR: mammalian target of rapamycin
NF‐kB: nuclear factor‐kappa B
OC: ovarian cancer
p21: cyclin‐dependent kinase inhibitor 1
p53: TP53, tumor protein P53
p70S6K1: p70S6 kinase
PERK: protein kinase R‐like ER kinase
PGE2: prostaglandin E2
PI3K: phosphatidylinositol‐4,5‐bisphosphate 3‐kinase
RAF: rapidly accelerated fibrosarcoma
RAS: rat sarcoma
STAT3: signal transducer and activator of transcription 3
TGF‐β: transforming growth factor beta
VEGF: vascular endothelial growth factor

## 1 Introduction

Recent studies indicate that food components (phytochemicals) have significant anticarcinogenic activities. These contain phytoestrogens, phytosterols and flavonoids. Phytoestrogens have been displayed including isoflavonoids (soy‐based foods) and lignans (fruits, vegetables, grains, and seeds) to have estrogenic as well as antiestrogenic activity (McCann et al. 2003). Outcome of a meta‐analysis indicates that high consumption of isoflavonoids or soy‐based foods is related to a reduced risk of ovarian cancer (Hedelin et al. 2011; Myung et al. 2009). Another class of phytoestrogens, lignans, have lower estrogenic activity than isoflavonoids, but also have antiestrogenic and anticarcinogenic effects (Hedelin et al. 2011). Some epidemiological studies have examined the related to phytoestrogens and breast, endometrial, and ovarian cancer (Lee et al. 2022). Phytoestrogens, a group of compounds derived from soybeans, are structurally similar to mammalian 17β‐estradiol (E2) and mimic weak estrogenic activities (Liu et al. 2023; Qu et al. 2014; Song et al. 2024).

Ovarian cancer (OC) is known as the deadliest gynecological cancer in the Western world due to its late diagnosis. Approximately 300,000 new cases and 185,000 deaths are notified worldwide each year. Ovarian cancer includes a wide variety of different cancers and has conventionally been classified as epithelial ovarian cancers (90% cases), germ cell tumors (5%), and sex cord‐stromal tumors (2%–5%) (Langdon et al. 2020). The etiology of ovarian cancer is not yet known, but established risk factors include genetic mutation, menopausal hormone use, obesity, and smoking (Song et al. 2024). Furthermore, ovarian cancer is thought to be a hormone‐sensitive tumor because approximately 60%–100% of tumors express estrogen receptors (ERs). There are two ER subtypes (ER‐α and ER‐β) (Chan et al. 2018). Expression of ER‐β in ovarian cancer cells has inhibited functionally cell proliferation and motility, and induced apoptosis (Bossard et al. 2012; Chan et al. 2018).

The genetic and molecular mechanisms underlying ovarian cancer are mostly unknown, and treatment options for patients with advanced disease are restricted. At the same time, investigation on ovarian cancer and the improvement of novel treatments have been hindered by the absence of suitable animal models (Eilati et al. 2013). In current narrative review, we provide information about elucidating the estrogen signaling mechanisms, and studying the possible targets of phytoestrogens in ovarian cancer.

## 2 Phtyoestrogens: Definition, Sources, Classification

Phytoestrogens are a large group of natural compounds found in over 300 plants. Phytoestrogens are naturally occurring, non‐steroidal polyphenolic plant‐derived compounds that are similar in structure to endogenous estrogens, which are human sex hormones (Lee et al. 2022; Petrine and Del Bianco‐Borges 2021; Rietjens et al. 2017). They are known as “dietary estrogens” due to their structural and functional similarity to the endogenous (17‐β estradiol) estrogen present in all mammals (Rietjens et al. 2017; Swathi Krishna et al. 2022), which allows them to bind to both estrogen receptors (ER), ER‐α and ER‐β, resulting in weak estrogenic activity. Phytoestrogens have been identified as antioxidant, antiinflammatory, antithrombotic, antiallergic, and antitumoral agents (Lee et al. 2022; Torrens‐Mas and Roca 2020).

Phytoestrogens are known to be found in fruits, vegetables and whole grains that are frequently consumed by humans as shown in Table 1 (Lephart 2021; Sirotkin and Harrath 2014; Swathi Krishna et al. 2022). Phytoestrogens are abundant in various edible and/or medicinal plants, mostly belonging to the *Leguminosae* family (Liu et al. 2023; Sirotkin and Harrath 2014). Phytochemicals with potential estrogenic activities contain soy, red clover, kudzu, hops, licorice, rhubarb, yam, and chasteberry (Hajirahimkhan et al. 2013).

**TABLE 1:** Phytoestrogen sources (Lephart 2021; Swathi Krishna et al. 2022).

Phytoestrogens	Sources
Flavonoids	
Isoflavones	Soybeans (soy food products, Asian foods, especially fermented foods), beans, peas, chickpeas, lentils, cabbage, lettuce, kale, peanuts, fruits, vegetables, onions, alfalfa, clover, spices, grains (wheat, barley, bran), cereals, seeds, kudzu, cow's milk, and eggs
Flavonols	Apples, cherries, berries, peppers, cruciferous vegetable (broccoli, cabbage, and sprouts), spinach, kale, tea, herbs, tomatoes, citrus fruits, cocoa, cranberries, whole grains, asparagus, red wine, capers, onions, olive oil, and legumes
Prenyl flavonoids	Hops, beer
Lignans	Flaxseed, linseeds, lentils, beans, peanuts, seeds/nuts, soybean, soy, chickpea, clover, rapeseed, sesame, whole‐grain cereals such as wheat, oats, rye and barley, legumes, various vegetables (like carrots and broccoli), fruits (especially berries), dairy products, meat, fish, and seaweed
Coumestans	Clover, alfalfa, and soybean sprouts, spinach, and lentils
Stilbenes	Grapes, berries, blue‐berries, raspberries, cranberries, mulberries, peanuts, and cocoapowder

**FIGURE 1:**
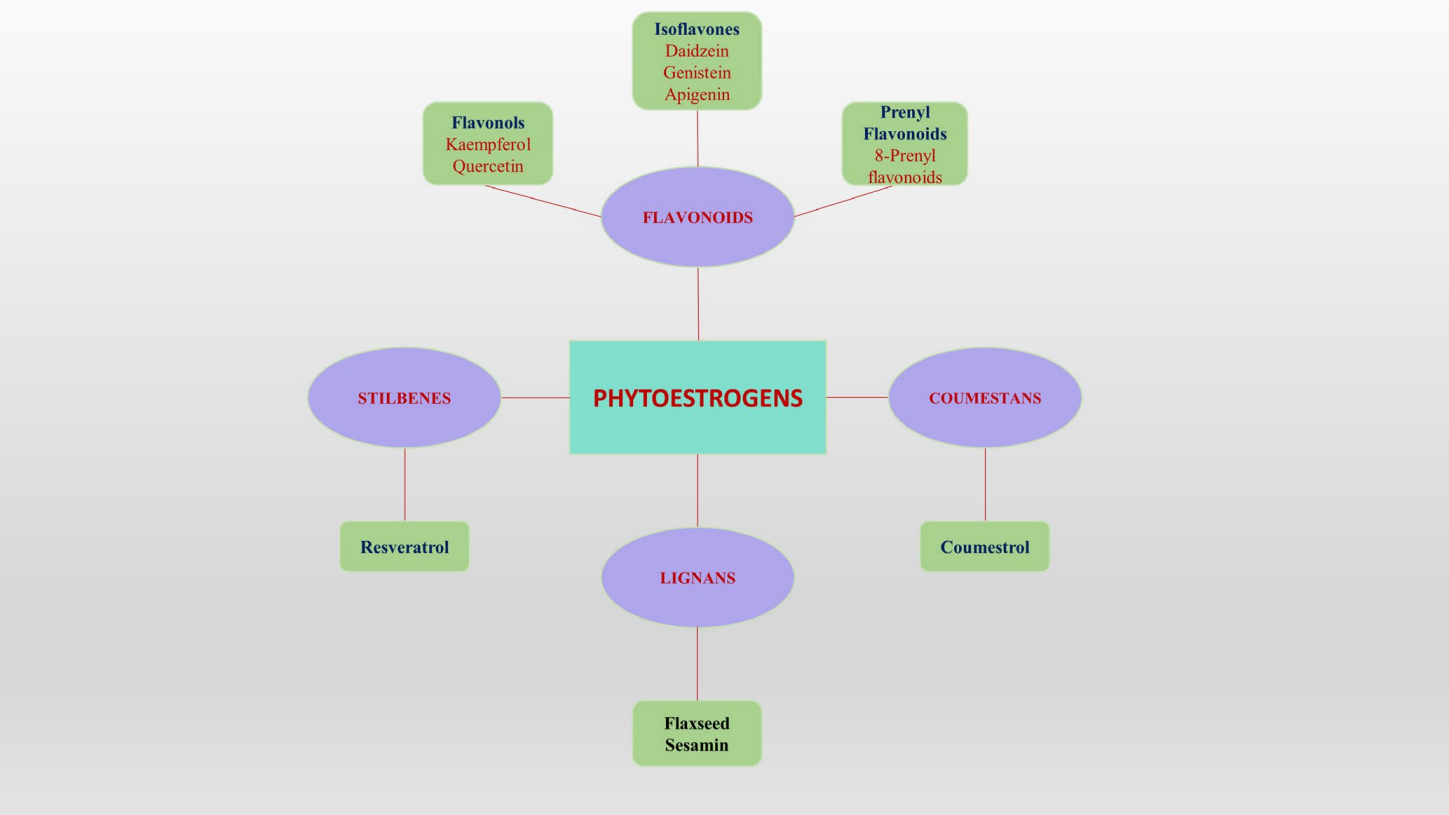
Phytoestrogens classification.

They are classified into four major groups: lignans, which are present in plant cell walls and fiber rich foods, and flavonoids (flavonols, isoflavones, which are principally located in soy crops, and prenylflavonoids), coumestans and stilbenes as represented in Figure 1 (Swathi Krishna et al. 2022). Isoflavones are present in legumes, mostly soybeans, flaxseed is a major source of lignans, and coumadins are found in significant amounts in clover, alfalfa, and soybean sprouts. 8‐Prenyl flavonoids are common in vegetables, hops, and beer (Sirotkin and Harrath 2014) (Table 1).

The major isoflavones include genistein, daidzein, equol, glycitin, formononetin, and biochanin A, which are mostly present in conjugated forms (genistin, daidzin, puerarin, glycitein, ononin, and sissotrin) in soy, soy‐based foods, and legumes (Patra et al. 2023; Rietjens et al. 2017). Genistein and daidzein and their metabolite equol are the most studied isoflavones (Patra et al. 2023). Flavones include kaempferol and quercetin. Lignans include enterolactone, enterodiol and nordihydroguaiaretic acid and coumestans are coumestrol (Qu et al. 2014).

## 3 Estrogen Signaling in Ovary and Ovarian Cancer

Preclinical and clinical studies suggest that estrogen plays an important role in the development and progression of some ovarian cancers (Langdon et al. 2020; Mungenast and Thalhammer 2014; Sarwar et al. 2022; Song et al. 2024).

Estrogen signaling occurs through various estrogen receptor isoforms. The so‐called genomic signaling is further enhanced by non‐genomic GPER‐1 (GPR30, GPER), a membrane‐bound G‐protein‐coupled receptor that can mediate both fast and transcriptional events in response to estrogen. Ovarian cancer cells express both estrogen receptor subtypes (ER‐α and ER‐β). Estrogen receptor alpha (ER‐α) is the primary mediator of the estrogen response. Several mutated forms of ER‐α and ER‐β have been identified in ovarian carcinomas (Langdon et al. 2020). GPER‐1 (G protein‐coupled estrogen receptor 1, GPR30, GPER) is a membrane‐enclosed G protein‐coupled receptor that binds estrogen and activates multiple downstream signaling pathways (Langdon et al. 2020; Prossnitz et al. 2008). GPER‐1 role in ovarian cancer has been somewhat controversial, with different studies showing conflicting results. Ignatov and colleagues have suggest that GPER‐1 plays a tumor suppressor role in ovarian cancer. Its expression has been notified to be lower in ovarian cancers than in benign or nonmalignant tissues. Furthermore, its expression has been notified to be higher in early‐stage cancers than in late‐stage cancers. Consistent with an inhibitory role, G1, a selective GPER‐1 agonist, inhibited the proliferation of ovarian cancer cell lines (SKOV‐3 and OVCAR‐3) (Langdon et al. 2020).

While ER‐α promotes growth and migration, ER‐β is reported to function primarily in growth inhibition. However, this has been stated to depend on the nature of the ER‐β isoforms present. Knowledge of the role and effect of GPER‐1 is still limited, with evidence for both tumor‐promoting and tumor‐suppressing roles, and its importance relative to ER‐α and ER‐β is unknown (Langdon et al. 2020).

**FIGURE 2:**
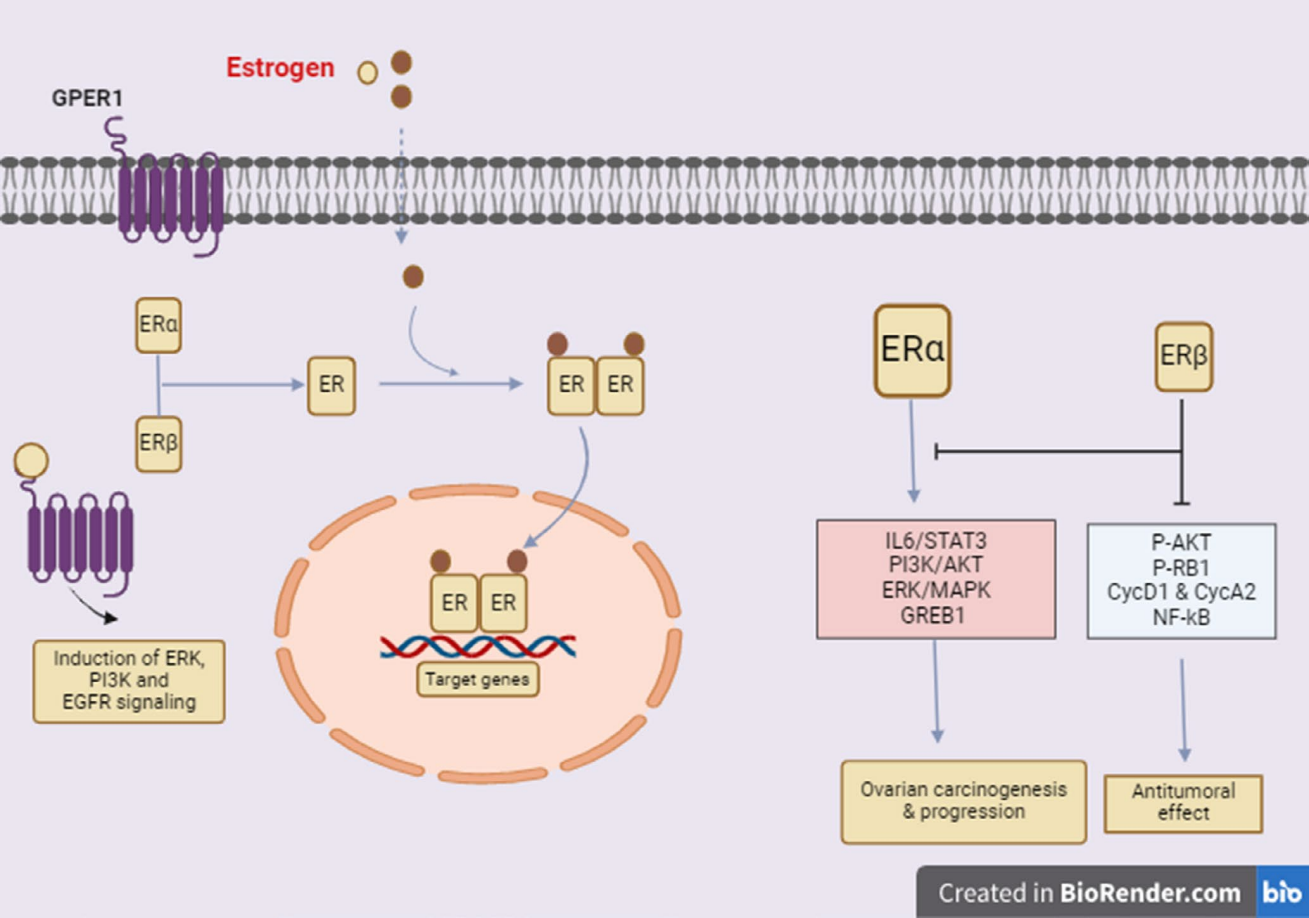
Main mechanisms of estrogen signaling in the tumorigenesis of ovarian cancer.

At the cellular level, the effects of estrogen on tumorigenesis are receptor‐dependent and receptor‐independent. Estrogen receptor‐α (ER‐α) causes transcriptional expression of estrogen‐responsive genes (proto‐oncogenes such as c‐fos, c‐myc, and HER2/neu) that provide signaling systems for cell division and differentiation. In addition to these genes, cell cycle regulators, cyclins, growth factors, and others are also involved. The membrane‐bound G‐protein‐coupled estrogen receptor (GPER) activates second messenger systems. Thus, GPER causes rapid nongenomic effects of estrogens (Mungenast and Thalhammer 2014). A study in endometrial cancer cells has shown that GPER mediates estrogen‐stimulated induction of ERK‐1/MAPK and −2 and phosphatidylinositol‐4, 5‐bisphosphate 3‐kinase (PI3K)/AKT, followed by transactivation of the epidermal growth factor receptor (EGFR) as represented in Figure 2 (Kozieł and Piastowska‐Ciesielska 2023; Mungenast and Thalhammer 2014; Petrie et al. 2013).

A group of researcher reveal that GPER is present in various tumor cells and its subcellular localization varies in the cell membrane, endoplasmic reticulum membrane, mitochondrial membrane, Golgi apparatus and nucleus of tumor‐associated fibrocytes. For example, in a breast cancer cell, GPER is expressed intensely in the cytoplasm but not in the nucleus. Apart from these, the current lack of three‐dimensional protein structure of GPER prevents detailed study of GPER protein structure (Huang et al. 2023). Therefore, to clarify this apparent dichotomy regarding the context‐dependent role of GPER, we defend that the specific cell type, tumor microenvironment, presence of other receptors, and specific ligands may determine whether GPER signaling promotes or inhibits tumor activity.

In another study, ER‐α has activated downstream pathways crucial for carcinogenesis, such as IL‐6/STAT3, PI3K/AKT, MAPK signaling, and pro‐invasive pathways as shown in Figure 2 (Borella et al. 2023). Hodgkinson and colleagues demonstrated the role of GREB‐1 (growth regulation in breast cancer by estrogen) in epithelial ovarian cancer as a promoter of tumor development and growth and as a possible co‐factor of ER‐α in the transcription of ERE genes as shown in Figure 2 (Borella et al. 2023). Additionally, Bossard et al. also demonstrated that ERβ downregulates protumoral factors such as P‐AKT, P‐RB1, CycD1, and CycA2 in BG‐1 (ERα‐positive) and PEO14 (ERα‐negative) cell lines transfected with ESR2 adenoviruses. All results were confirmed in mouse models. Also, Liu et al. used RNA‐seq analysis to show that ER‐β can alter the expression of several protumoral genes when activated by an agonist. They indicate that ER‐β inhibits NF‐κB via a non‐canonical interaction with its subunit p‐65 and that ER‐β increases the sensitivity of chemotherapy‐resistant epithelial ovarian cancer cell lines as shown in Figure 2 (Borella et al. 2023).

## 4 Possible Targets of Phytoestrogens in Ovarian Cancer: Preventive and Curative, Aggression

Ovarian cancer (OC) is characterized by rapid growth, high metastasis, and quick drug resistance, which is the most common cancer in women (Swathi Krishna et al. 2022). Phytoestrogens mimic or antagonize naturally occurring endogenous estrogens, which are a group of plant‐derived compounds, to promote or inhibit estrogenic responses. Phytoestrogens can bind to estrogen receptors (ERs) due to being chemically similar to estradiol (E2, 17β‐estradiol). Phytoestrogens show a much weaker ER‐binding affinity than estradiol and have a variety of roles in the regulation of the reproductive system (Qu et al. 2014). Although phytoestrogens can bind to both α‐ and β‐estrogenic receptors, they have a greater affinity for β‐receptors. They bind to β‐receptors and act as agonists, partial agonists, and antagonists (Mottaghi and Abbaszadeh 2022).

Low‐grade serous ovarian cancer has been shown to be mediated through activation of the MAPK pathway via RAS/RAF, and has been associated with high levels of estrogen receptor and progesterone receptor expression. However, another study has reported that ovarian cancer cells SKOV3 (ER‐sensitive) undergo apoptosis via cell cycle arrest, which has been mediated through GPER (Sarwar et al. 2022).

**FIGURE 3:**
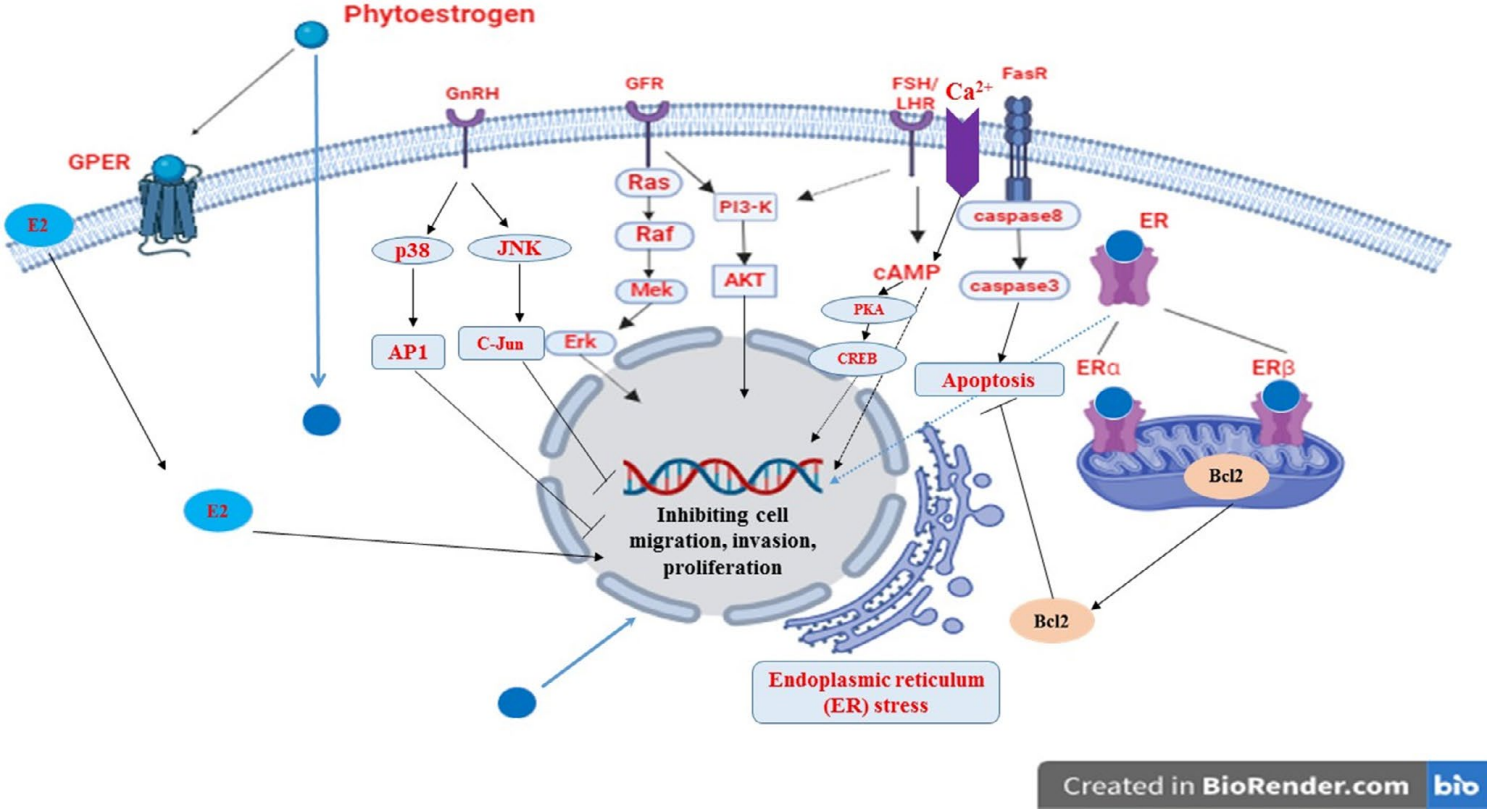
Schematic representation of possible signal pathways of phytoestrogens in ovarian cancer.

Studies in various in vitro and in vivo models suggest that phytoestrogens may act on signaling pathways such as ER (endoplasmic reticulum)‐dependent signaling, GnRH receptor, FSH or LH receptors and hormones, and GFR, which help to regulate the expression of Akt, Raf, caspase‐3, NF‐kB, and Bcl‐2. This induces apoptosis in ovarian cancer by inhibiting metastasis and cell proliferation as represented in Figure 3 (Dull et al. 2019; Hwang et al. 2013; Swathi Krishna et al. 2022).

Current studies have indicated that the beneficial effect of phytoestrogens in the management of ovarian cancer (Swathi Krishna et al. 2022). Genistein has been studied extensively on ovarian cancer cells (Lee et al. 2012). Genistein and daidzein (10 and 50 μM) have induced apoptosis by altering FAK and PI3K/AKT/GSK signaling pathways and p21/cyclin D1 expression in ovarian cancer cell lines. These phytoestrogens have prevented cell migration, invasion, and proliferation, and have caused cell cycle arrest (Chan et al. 2018; Swathi Krishna et al. 2022).

Genistein and daidzein are displayed higher affinity to bind ER‐β. Estrogen receptor modulators, genistein and daidzein, inhibit cell migration, invasion, proliferation, and sphere formation in ovarian cancer via modulation of FAK and PI3K/AKT signaling (Chan et al. 2018). Phytoestrogen genistein has inhibited ovarian oxidative damage and apoptotic cell death‐induced by ionizing radiation by promoting ER‐β, TGF‐β, and FOXL‐2. Genistein has hindered ovarian apoptosis through upregulation of ER‐β and FOXL‐2 with downregulation of TGF‐β expression. Genistein could ensure a novel therapeutic modality for protecting ovarian function of female who have survived ovarian cancer (Haddad et al. 2020). Additionally, genistein may inhibit the growth of ovarian cancer cells through the regulation of cell growth‐related genes such as caspase‐3, Bcl‐2, and VEGF as represented in Table 2 (Qu et al. 2014). Genistein has indicated upregulation of cyclin D1 and CDK4 expression, thereby promoting the proliferation and viability of ovarian cancer OVCAR‐5 cells (Wang et al. 2021). Genistein has prevented epithelial–mesenchymal transition and migration capacities of BG‐1 ovarian cancer cells enhanced by E2 (17β‐estradiol) via ER signaling and the downregulation of TGF‐β signal as represented in Table 2 (Kim et al. 2015).

**TABLE 2:** Phytoestrogens and targeting signal pathways in ovarian cancer cells.

Phytoestrogens	Targets	Cells/models	References
Genistein and Daidzein	FAK and PI3K/AKT/GSK signaling pathways and p21/cyclin D1 expression	SKOV‐3, A2780/CP70 and OVCAR‐3 cells	Chan et al. (2018) and Swathi Krishna et al. (2022)
Genistein	The inhibition of cell cycle progression	BG‐1 ovarian cancer cells	Hwang et al. (2013)
Genistein	Inhibited ovarian oxidative damage and apoptotic cell death‐induced by ionizing radiation by promoting up‐regulation of ER‐β and FOXL‐2 with downregulation of TGF‐β	Three‐week‐old female Sprague–Dawley rats	Haddad et al. (2020)
Genistein	The regulation of cell growth‐related genes such as caspase‐3, Bcl‐2, and VEGF	A2780 and its PR clone C200 cells OVCAR‐3 cells	Solomon et al. (2008) and Luo et al. (2008)
Genistein	Indicated upregulation of cyclin D1 and CDK4 expression, thereby promoting the proliferation and viability of ovarian cancer	OVCAR‐5 cells	Wang et al. (2021)
Genistein	Prevented EMT and migration, enhancing by E2, via ER signaling and the downregulation of TGF‐β signal. Apoptosis (caspase‐3/7 activity)	BG‐1 ovarian cancer cells	Kim et al. (2015)
Genistein	Microtubule depolymerization	SKOV‐3, ES2, HeyA8, HeyA8‐MDR cells	Ahmed et al. (2011)
Resveratrol	Akt/GSK3 by inhibiting protein glycosylation	PA‐1 (p53 wild type), MDAH2774 (p53 mutant) and SKOV3 (p53 null) cells	Gwak et al. (2016)
Resveratrol	Inactivated STAT3 signaling	OC‐CAOV‐3 and OVCAR‐3 cells	Zhong et al. (2018)
Resveratrol	Down‐regulated Akt/GSK and ERK signalling pathways	OVCAR‐3 cells	Vergara et al. (2012)
Resveratrol	Inhibited rat orthotopic ovarian cancer growth without affecting normal tissues by diminishing STAT3 expression	NUTU‐19 OC	Zhong et al. (2019) and Xu et al. (2021)
Lignan (flaxseed)	Showed potential in effective treatment and prevention of ovarian cancer by targeting inflammatory prostaglandin pathways	Three hundred eighty seven single comb 2.5 year old White Leghorn hens	Eilati et al. (2013)
Lignan and omega‐3 fatty acids	Increased activation of p38 and ERK 1/2 MAPK and increased apoptosis in tumor epithelium	BG1FR human ovarian adenocarcinoma cell lines and two and half year old hens (*Gallus domesticus*)	Dikshit et al. (2017)
2‐Methoxyestradiol	Induced apoptosis via p38‐MAPK pathway	BG1, HeyC2, and TOV112D human ovarian cancer cells, two and half year‐old White leghorn Hy‐line W‐36 chickens	Pal et al. (2019)
Apigenin	Inhibition of IL‐6/STAT3 signaling pathway and downregulation Axl and Tyro3 receptor tyrosine kinases	Taxol‐resistant SKOV3/TR cells	Suh et al. (2014)
Apigenin	Suppressed PI3K/AKT/mTOR signaling pathway by regulating the expression level of ERα/ERβ	OVCAR‐3 cells	Liu et al. (2021)
Apigenin	Inhibited proliferation of ovarian cancer A2780 cells through ATF3/Id1 pathway	A2780 and OVCAR‐3 cells	Li et al. (2009)
Apigenin	Inhibited expression of VEGF at the transcriptional level through expression of HIF‐1 via the PI3K/AKT/p70S6K1 and HDM2/p53 pathways	A2780 and OVCAR3 cells	Fang et al. (2005) and Patel et al. (2007)
Apigenin	Inhibited formation of tube by endothelial cells in vitro and the activity of MAPK and PI3K	HO‐8910PM cells	Patel et al. (2007) and Zhu et al. (2003)
Kaempferol	Showed major inhibitor effects on angiogenesis and on VEGF gene expression through both Akt/HIF and ESRRA pathways	OVCAR‐3 (mutant p53), wild type p53 of A2780/CP70 cells	Amjad et al. (2022)
Kaempferol	Induced G2/M cell cycle arrest through Chk2/Cdc25C/Cdc2 and Chk2/p21/Cdc2 pathways Enhanced the expression level of DR5 and Fas and induce the extrinsic apoptosis pathway through death receptors/FADD/Caspase‐8	A2780/CP70 ovarian cancer cells	Amjad et al. (2022) and Gao et al. (2018)
Kaempferol	Induced autophagy and apoptosis G0/G1 cell cycle arrest and inhibition of MEK/ERK and STAT3 pathways	Caov‐3, TOV‐112D, SKOV‐3, and OVCAR‐3 cells	Amjad et al. (2022) and Yang et al. (2019)
Quercetin	Induced protective autophagy and apoptosis through ER stress via the p‐STAT3/Bcl‐2 axis	Ovarian cancer mice xenograft model	Liu et al. (2017), Shafabakhsh and Asemi (2019) and Vafadar et al. (2020)
Quercetin	Inhibited cell growth and induced apoptosis by decreasing Bcl‐2, Bcl‐xL and boosting caspase‐3, caspase‐9, cyto‐c, Bid, Bad, and Bax expression levels	PA‐1cells	Shafabakhsh and Asemi (2019), Teekaraman et al. (2019) and Vafadar et al. (2020).
Quercetin	Induced protective autophagy and apoptosis via ER stress by the p‐STAT3/Bcl‐2 axis	SKOV‐3 cells	Ren et al. (2015) and Vafadar et al. (2020)
Quercetin	Inhibited survival and proliferation via inactivating PI3k/Akt, Ras/Raf pathways and EGFR expression	PA‐1 cells	Dhanaraj et al. (2021)

Resveratrol induces ER (endoplasmic reticulum) stress‐mediated apoptosis in cancer cells involving Akt/GSK3 by inhibiting protein glycosylation in cancer cells. Resveratrol also aids hexosamine biosynthesis to trigger the degradation of protein glycosylation (Gwak et al. 2016). Resveratrol has targeted multiple oncogenic and oncosuppressive signaling pathways in ovarian cancer prevention and treatment. Resveratrol has been reported to induce apoptosis in human ovarian cancer cell lines, leading to compromised cell proliferation (Xu et al. 2021). Resveratrol has inhibited ovarian tumor growth in an in vivo mouse model. Resveratrol has been shown to have significant antineoplastic activity and suppressed glucose metabolism in a xenograft mouse model of ovarian carcinoma. Furthermore, resveratrol has inhibited tumor cell growth after treatment with cisplatin both in vitro and in vivo. These results suggest that resveratrol has therapeutic potential in the treatment of epithelial ovarian carcinoma (Tan et al. 2016). Resveratrol has down‐regulated the protein cyclin D1 and the phosphorylation levels of protein kinase B (Akt) and glycogen synthase kinase‐3b (GSK‐3b) in a concentration‐dependent manner. Dephosphorylation of these kinases may be responsible for the decreased cyclin D1 levels observed after treatment. Additionally, resveratrol has been shown to reduce the phosphorylation levels of extracellular signal‐regulated kinase (ERK) 1/2. Furthermore, resveratrol has shown inhibitory effects on AKT phosphorylation in cultured cells derived from ascites of ovarian cancer patients and in a panel of human cancer cell lines (Vergara et al. 2012).

Phytoestrogen lignans (flaxseed) have shown potential in effective treatment and prevention of ovarian cancer by targeting inflammatory prostaglandin (PG) pathways. Cyclooxygenase (COX) acts as the rate‐limiting enzyme in the production of prostaglandins (PG). COX has two isoenzymes: COX‐1 and COX‐2. High expression of COX‐1 has been shown in ovarian cancer. Apart from this, Prostaglandin E2 (PGE2) is the most common prostaglandin in various human cancers such as colon, lung, breast, and head and neck cancers and acts an significant target in cancer so that inhibition prostaglandin pathways has been indicated as a possible target to prevention ovarian cancer (Eilati et al. 2013). The effects of lignan and omega‐3 fatty acids, two main components of flaxseed, on estrogen metabolism and estrogen receptor in ovarian tumors have been investigated. ER alpha expression has been upregulated in ovarian tumors. Increased activation of p‐38 and ERK‐1/‐2 MAPK and increased apoptosis in tumor epithelium have been observed (Dikshit et al. 2017). 2‐Methoxyestradiol and docosahexaenoic acid, the biologically derived active compounds from flaxseed diet have been investigated. 2‐Methoxyestradiol induces apoptosis via p38‐MAPK pathway in ovarian tumors as represented in Table 2. Furthermore, they decrease angiogenesis in ovarian tumors but not in normal ovarian tissues (Pal et al. 2019). Flaxseed has been proven to reduce the severity of ovarian cancer by protecting women from ovarian tumor metastasis by reducing omental fat (Weston et al. 2021).

Apigenin has inhibited proliferation of ovarian cancer A2780 cells through ATF3/Id1 pathway (Id1: inhibitor of differentiation or DNA binding protein 1) as represented in Table 2 (Li et al. 2009). Fang et al. have shown that apigenin inhibits expression of VEGF in human ovarian cancer cells. Apigenin has inhibited VEGF expression at the transcriptional level through expression of HIF‐1 via the PI3K/AKT/p70S6K1 and HDM2/p53 pathways. Apigenin has also inhibited formation of tube by endothelial cells in vitro. In addition, apigenin has inhibited the activity of MAPK and PI3K in human ovarian carcinoma HO‐8910 PM cells (Patel et al. 2007). Apigenin has displayed the potential treatment to circumvent taxol‐resistance in SKOV‐3 ovarian cancer cells (Suh et al. 2014). Also, apigenin prevented proliferation of ovarian cancer cells via histamin‐induced by downregulating ER‐α/ER‐β expression. Apigenin has downregulated ER‐mediated PI3K/AKT/mTOR expression (Liu et al. 2021).

Many studies have shown that kaempferol has the potential to induce apoptosis of ovarian cancer cells, inhibiting cell proliferation, and triggering autophagy, preventing metastasis and invasion (Ma et al. 2023). According to the study on OVCAR‐3 (mutant p53), wild type p53 of A2780/CP70 cells, kaempferol has showed major inhibitor effects on angiogenesis and on VEGF gene expression through both Akt/HIF and ESRRA (estrogen related receptor alpha) pathways (Amjad et al. 2022). Another study has verified the tumor antiangiogenesis effects of kaempferol and this flavonoid compound has induced G2/M cell cycle arrest through Chk2/Cdc25C/Cdc2 and Chk2/p21/Cdc2 pathways in A2780/CP70 ovarian cancer cells. The investigators have explained that kaempferol could enhance the expression level of DR5 and Fas and induce the extrinsic apoptosis pathway through death receptors/FADD/Caspase‐8 and Chk2, and have indicated that p53 is not a factor responsible for apoptosis induction and upregulation by kaempferol (Amjad et al. 2022; Gao et al. 2018). Kaempferol has also showed antiproliferative effects in various human ovarian cancer cells (Caov‐3, TOV‐112D, SKOV‐3, and OVCAR‐3) by inducing autophagy and apoptosis G0/G1 cell cycle arrest and inhibition of MEK/ERK and STAT3 pathways (Amjad et al. 2022; Yang et al. 2019). According to a recent study on A2780 cells, kaempferol has been found to increase apoptosis and decrease survival and proliferation. Additionally, increased levels of GRP78, PERK, ATF6, IRE‐1, LC3II, beclin‐1, and caspase‐4 have induced the cytotoxic endoplasmic reticulum/autophagy mechanism, which has been associated with increased intracellular calcium cation levels. Kaempferol has downregulated p‐Akt protein in cancer cells, making them more sensitive to cisplatin. To be noted, nanoformulations of kaempferol may reduce the survival rate of ovarian cancer cells more effectively than kaempferol alone (Amjad et al. 2022).

Quercetin has induced autophagy and apoptosis through ER (endoplasmic reticulum) stress via the p‐STAT3/Bcl‐2 axis in ovarian cancer SKOV‐3 cells (Liu et al. 2017; Ren et al. 2015; Shafabakhsh and Asemi 2019; Vafadar et al. 2020). Quercetin has inhibited human metastatic ovarian cancer cell growth and induced apoptosis by decreasing Bcl‐2, Bcl‐xL and boosting caspase‐3, caspase‐9, cyto‐c, Bid, Bad, and Bax expression levels in PA‐1cells (Shafabakhsh and Asemi 2019; Teekaraman et al. 2019; Vafadar et al. 2020). Quercetin has inhibited survival and proliferation via inactivating PI3k/Akt, Ras/Raf pathways and EGFR expression in human metastatic ovarian cancer PA‐1 cells. In addition, quercetin has also decreased the secretion of gelatinase enzyme, proteolytic activity of MMP‐2/‐9, and both MMPs gene expression in metastatic ovarian cancer PA‐1 cells. Furthermore, quercetin has inhibited the migration of PA‐1 cells (Dhanaraj et al. 2021).

## 5 Discussion

Ovarian cancer is often diagnosed at a late stage when the cancer has spread to other organs. So far, there are only a limited number of options for successful chemotherapy treatment, and new strategies are needed. Several epidemiological and experimental data have suggested that ovarian cancer has many estrogen‐promoted pathways in common with other hormone‐related cancers such as breast cancer (Mungenast and Thalhammer 2014). Ovarian cancer cells express both estrogen receptor subtypes (ER‐α and ER‐β), which exert opposite effects on carcinogenesis. Recently, targeting ER‐β in diverse cancers has received significant attention due to the antitumorigenic effects of ER‐β (Chan et al. 2018).

Phytoestrogens exhibit a molecular structure similar to endogenous estrogen and estradiol. The tendency of these phytoestrogens to bind to estrogen receptors on different cell types and produce estrogenic or antiestrogenic effects is due to their chemical similarity to their endogenous counterparts (Swathi Krishna et al. 2022). Genistein, a widely studied isoflavone, has been shown to have a slightly greater reduction in ovarian cancer risk, similar to in vitro studies and some in vivo animal studies (Qu et al. 2014). Some in vitro assays have been performed with receptor binding studies and cell proliferation assays using ovarian BG‐1 cells obtained from female estrogen‐sensitive tissues to detect estrogen activity (Rietjens et al. 2017). Due to its estrogen‐like properties, in vitro studies have indicated an inverse relationship between phytoestrogen intake and ovarian cancer risk in cell lines like SKOV‐3 cells, OVCAR‐3 cells, and cell lines from patients. Additionally, an in vivo study has verified that genistein has an important role in antitumor activity in dimethylbenz[a]anthracene (DMBA)‐induced ovarian cancer in female Sprague–Dawley rats (Qu et al. 2014). In light of this information, it could be said that phytoestrogens exhibited inhibitory properties in in vitro studies, while their therapeutic effects were observed in in vivo studies. In addition, the prevalence of in vitro data is greater than that of in vivo data. More in vivo research is needed.

Qu et al. have shown that higher phytoestrogen intake has a potential protective effect against ovarian cancer compared to lower phytoestrogen intake. They have indicated that isoflavones may act the most significant role in protective effects of phytoestrogen. The dose of phytoestrogen intake has been particularly large between Asians and non‐Asians. The results have showed that phytoestrogens have a significant protective effect in Asians but not in non‐Asians (Qu et al. 2014). Furthermore, epidemiological studies indicate that soy isoflavon intake reduces the risk of ovarian cancer in China and Japan (Chan et al. 2018). As a result, beneficial phytoestrogen intake habits in Asians has provided a great advantage for their protective effects. This study should be confirmed in future larger, well‐designed observational studies.

Another study has investigated the association between a diabetes risk‐reducing diet and ovarian cancer using data from a multicenter Italian study. According to the results obtained, high compliance with the diet that reduces the risk of diabetes has been found to be inversely proportional to ovarian cancer (Esposito et al. 2023). As a result, this work indicates that high adherence to a diet that can reduce the risk of diabetes could be inversely related to ovarian cancer. It has been emphasized that more evidence from prospective studies is needed to support our findings.

A Swedish cohort study has found no relation between dietary phytoestrogens and the risk of ovarian cancer; however, the Swedish diet is possible to include low amounts of phytoestrogens from nuts, berries, beans/soy, and whole‐grain bread, complicating the determination of a relation (Hedelin et al. 2011). As a result, no association has been found between the overall risk of ovarian cancer and the intake of specific food items rich in isoflavonoids, lignans, and coumestrol, phytoestrogens, or fiber. The findings indicate that phytoestrogens do not have a significant etiological role in ovarian cancer in a cohort of Caucasian women with a low bean/soy diet. With this, further studies are needed to evaluate the differences in dietary effects between invasive and borderline ovarian cancer.

A meta‐analysis of 19 observational studies has found that fiber intake has been inversely associated with the risk of ovarian cancer (Xu et al. 2018). Dietary fiber intake has reduced the risk of ovarian cancer. It will be necessary to test the relationships between different types of fiber (including soluble, insoluble, vegetable, fruit, grain, and legume fiber) on ovarian cancer.

The literature demonstrates that, after 3 year of treatment, the phytoestrogen genistein has exhibited a promising safety profile in reproductive tissues with positive effects on bone formation in a cohort of postmenopausal women (Marini et al. 2008). Indeed, it has been suggested that the rational intake of nutraceuticals (including isoflavones), particularly abundant in plant‐based eating patterns could be very useful in the prevention and cure of noncommunicable diseases without harmful side effects on thyroid and reproductive tissues, including breast tissue, on the possible therapeutic role of isoflavones in combination with an healthy diet (i.e., a plant‐based diet, as “Mediterranean diet”) and an adequate physical activity, particularly in post‐menopausal women (Marini 2022). Current evidence suggests that the gut microbiota is an integral regulator of estrogen status with clinical relevance to women's health and hormonal disorders, also modulating the therapeutic effects of diet/nutraceuticals/drugs (Choi &Choi, Choi and Choi 2024).

Genistein and daidzein are considered phytoestrogens that can mimic the binding of estrogen to ERs with higher affinity for ER‐β than ER‐α, which are the two main isoflavones found in soybean products and are also commercially available. Genistein has prevented PI3K/AKT phosphorylation, increasing expression of p21. These results suggest that inactivation of PI3K/AKT signaling associated with p21 induction acts a significant role in genistein‐induced G2/M arrest in ovarian cancer. AKT activation also has inactivated GSK3β via phosphorylation of Ser9 (Chan et al. 2018). Another study has also shown that genistein induces apoptosis in ovarian cancer via suppression of AKT signaling (Lee et al. 2012). A study indicates that genistein could be a new treatment option for protecting ovarian function in female cancer patients (Haddad et al. 2020). According to epidemiological and experimental studies, genistein has shown potential as a chemopreventive agent. Genistein has affected numerous cellular signaling pathways such as PTK, Akt, NF‐κB, MMP, and Bax/Bcl‐2. Although there are many studies on genistein in various cancers, there is no registered clinical trial on ovarian cancer. The role of genistein in ovarian carcinogenesis needs to be further investigated (Chan et al. 2018).

Resveratrol is the polyphenol compound with the most extensive evidence supporting its anticancer properties. In animal models, the toxicity of resveratrol has helped to advance its use in clinical studies. It has been suggested that it may be a useful complementary medicine adjunct for the prevention and treatment of ovarian cancer. In addition, randomized clinical trials should be conducted to define the therapeutic efficacy of resveratrol in ovarian cancer. The effects of using resveratrol in combination with conventional chemotherapy, targeted therapy, or immunotherapy should be further investigated. New, more effective compounds that mimic the effects of resveratrol should be developed (Xu et al. 2021).

Apigenin, phytoestrogen, slowed the growth of many types of tumors, but its antitumor mechanism is not yet clear. However, it has inhibited the proliferation of ovarian cancer cells. Apigenin has estrogen‐like properties. Its mechanism has showed that it has a certain regulatory effect on the expression of ERs. Apigenin has affected the PI3K/AKT/mTOR signaling pathway by promoting ER‐α/ER‐β expression, slowing tumor development by histamine. Nevertheless, apigenin's molecular mechanisms should be further researched (Liu et al. 2021).

Kaempferol has been observed to have a highly effective anticancer potential against a number of malignant tumors. Several studies have shown that it has the potential to induce tumor cell autophagy in ovarian cancer. Since its anticancer mechanism is the PI3K/AKT pathway and ROS, it can also trigger apoptosis. It also regulates cyclin and CDK, thereby exhibiting antiproliferative activity. However, research on the effects of kaempferol on gynecological cancers is limited to in vitro studies, and fewer in vivo studies are available (Ma et al. 2023). Therefore, further preclinical studies on kaempferol are needed.

For ovarian cancer, only the combination of cisplatin and kaempferol has been successful in overcoming cisplatin resistance in OVCAR‐3 cells, while apigenin, genistein, and quercetin have been reported to have no effect. In conclusion, kaempferol as a dietary component has sensitized ovarian cancer cells to cisplatin treatment, and further study is warranted for its application in the chemotherapy of ovarian cancers (Torrens‐Mas and Roca 2020). It has also been reported that epigallocatechin gallate increases the antitumor activity of cisplatin in ovarian cancer cells (Torrens‐Mas and Roca 2020).

According to the results obtained, quercetin may prevent ovarian cancer through several mechanisms such as antiinflammation, prooxidation, antiproliferation, and cell cycle arrest. Moreover, this natural compound has the ability to enhance the effects of other chemotherapeutic drugs. Further studies should be conducted on this compound to reveal its detailed mechanisms of action in ovarian cancer. In addition to various advantages, the use of quercetin is restricted for therapy due to different limitations such as very poor bioavailability, poor absorption, rapid metabolism, chemical instability, and rapid systemic elimination. The use of quercetin analogs and targeting quercetin with nanomedicine can circumvent these restrictions. So far, no clinical studies have been conducted to evaluate in ovarian cancer the effects of quercetin (Huang et al. 2023; Vafadar et al. 2020). Similarly, resveratrol has problems such as solubility, poor stability, and low bioavailability, which make drug development difficult. We can overcome these shortcomings by using nanomaterial drug delivery technology or advanced dosage formulations to increase efficacy (Huang et al. 2023). Phytoestrogens will be the future focus of ovarian cancer to enhance their pharmacological effects using nanomedicine approaches and to investigate their interaction mechanisms with GPER and many other signaling pathways.

## 6 Conclusion

Phytoestrogen intake has been linked to a reduction in cancer incidence and phytoestrogens are shown to be promising chemopreventive compounds. In addition to interfering with estrogen signaling pathways and regulating gene expression, phytoestrogens are also powerful antioxidants, regulate normal protein activity, and modulate epigenetics. In this way, phytoestrogens have the potential to prevent cell proliferation in many cancer cells (Torrens‐Mas and Roca 2020). Researches indicate that phytoestrogens may be possible hormonal treatment options for ovarian cancer patients (Chan et al. 2018). Also, phytoestrogens could sensitize cancer cells to treatments of anticancer, such as hormone therapy, chemotherapy, and radiotherapy (Torrens‐Mas and Roca 2020). Some researches also indicate that phytoestrogens could protect healthy cells without affecting the effectiveness of the treatment. However, the signaling mechanisms in ovarian cancer and the signaling mechanisms of phytoestrogens are still not exactly understood.

This narrative review highlights the role of estrogen in ovarian cancer and an overview of estrogen signaling pathways in ovarian cancer. Furthermore, strategies to target signaling pathways via phytoestrogens in ovarian cancer are discussed. In light of the data obtained, more research is needed on phytoestrogen signaling pathways in ovarian cancer cells.

## Author Contributions

**Ozgur Kutuk:** conceptualization, investigation, writing – original draft, methodology, supervision, writing – review and editing. **Ayse Kaplan:** conceptualization, investigation, methodology, writing – original draft, supervision, writing – review and editing.

## Conflicts of Interest

The authors declare no conflicts of interest.

## Data Availability

Data sharing not applicable to this article as no datasets were generated or analysed during the current study.
